# Characterization of a New Trioxilin and a Sulfoquinovosyl Diacylglycerol with Anti-Inflammatory Properties from the Dinoflagellate *Oxyrrhis marina*

**DOI:** 10.3390/md15030057

**Published:** 2017-02-27

**Authors:** Eun Young Yoon, A. Reum Yang, Jaeyeon Park, Seung Joo Moon, Eun Ju Jeong, Jung-Rae Rho

**Affiliations:** 1 Environment and Resource Convergence Center, Advanced Institutes of Convergence Technology, Suwon 16229, Korea; journal04@snu.ac.kr (E.Y.Y.); bada0@snu.ac.kr (J.P.); sjmoon04@snu.ac.kr (S.J.M.); 2 School of Earth and Environmental Sciences, College of Natural Sciences, Seoul National University, Seoul 08826, Korea; 3 Department of Oceanography, Kunsan National University, San 68 Miryong-dong, Kunsan, JeonBuk 573-701, Korea; yar0241@naver.com; 4 Department of Agronomy & Medicinal plant Resources, Gyeongnam National University of Science and Technology, Jinju 660-758, Korea; ejjeong@gntech.ac.kr

**Keywords:** *Oxyrrhis marina*, trioxilin, sulfoquinovosyl diacylglycerols, nitric oxide inhibition, docosahexaenoic acid

## Abstract

Two new compounds—a trioxilin and a sulfoquinovosyl diacylglycerol (SQDG)—were isolated from the methanolic extract of the heterotrophic dinoflagellate *Oxyrrhis marina* cultivated by feeding on dried yeasts. The trioxilin was identified as (4*Z*,8*E*,13*Z*,16*Z*,19*Z*) -7(*S*),10(*S*),11(*S*)-trihydroxydocosapentaenoic acid (**1**), and the SQDG was identified as (2*S*)-1-*O*-hexadecanosy-2-*O*-docosahexaenoyl-3-*O*-(6-sulfo-α-d-quinovopyranosyl)-glycerol (**2**) by a combination of nuclear magnetic resonance (NMR) spectra, mass analyses, and chemical reactions. The two compounds were associated with docosahexaenoic acid, which is a major component of *O. marina*. The two isolated compounds showed significant nitric oxide inhibitory activity on lipopolysaccharide-induced RAW264.7 cells. Compound **2** showed no cytotoxicity against hepatocarcinoma (HepG2), neuroblastoma (Neuro-2a), and colon cancer (HCT-116) cells, while weak cytotoxicity was observed for compound **1** against Neuro-2a cells.

## 1. Introduction

Heterotrophic protists are known to be important food sources for zooplanktons in the marine environment; they provide essential fatty acids to zooplanktons and act as trophic intermediates between the microbial loop and higher trophic levels [1,2,3]. Among them, the heterotrophic dinoflagellate *Oxyrrhis marina* has been reported to be a typical efficient producer of long-chain essential fatty acids such as, eicosapentaenoic acid (EPA, 20:5, *n*-3) and docosahexaenoic acid (DHA, 22:6, *n-*3) [4,5]. The unusually and small-sized (15–40 μm) *O. marina* is commonly found in shallow waters as well as in littoral and supralittoral pools [6,7]. This species is so easy to isolate and culture in the laboratory that it could be used as a model species for a broad range of ecological and biogeographic studies.

When investigating the possibility of producing DHA-containing commercial oil through the massive culture of *O. marina*, we recently isolated two types of compounds derived from DHA: a trioxilin (which is a trihydroxy DHA), and a sulfoquinovosyl diacylglycerol (SQDG) with a DHA component. Most trioxilins are hydrolysis products of hepoxilins, which are epoxide derivatives of arachidonic acids (AA, C20:4, *n*-6) and EPA, metabolized by lipoxygenase [8,9]. It has been reported that trioxilins mediate acetylcholine-induced vasodilation in the rabbit aorta and activate the peroxisome proliferator-activated receptor alfa (PPARα) [10,11]. Though unusual conversion from DHA to trioxilins in the rat pineal gland and hippocampus has been identified [12], this is the first study to report the isolation and the complete chemical structure of a DHA-derived trioxilin from the marine dinoflagellate *O. marina*. The compound showed significant nitric oxide (NO)-inhibitory activity of this trioxilin on lipopolysaccharide (LPS)-induced RAW264.7 cells. Along with the trioxilin, the newly isolated SQDG comprised a glycerol with a docosahexaenoyl substituent. SQDGs have been reported to show inhibitory activity against DNA polymerase, HIV-reverse transcriptase type 1, and platelet-activating factor receptor binding [13,14]. Like digalactosyldiacylglycerols and other monogalacytosyl analogs isolated from microalgae, the SQDG isolated in our study also showed strong NO-inhibition in murine macrophage RAW264.7 cells [15,16], more significant than the trioxilin isolated from the same extract.

Here we will describe the structure determination of the two compounds, along with their isolation and biological activity.

## 2. Results and Discussion

**Figure 1:**
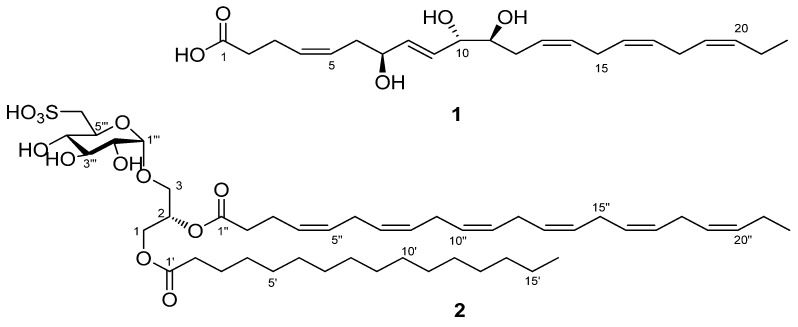
Chemical structures of compounds **1** and **2**.

The heterotrophic dinoflagellate *O. marina* was cultivated to a volume of 1000 L by feeding on dried yeasts in sea water. The MeOH extract from the harvested cells was fractionated using high-performance liquid chromatography (HPLC) with a size exclusion column, and the fractions were purified by reversed-phase HPLC to generate compounds **1** and **2** (Figure 1). Both the compounds were analyzed using a combination of NMR and mass spectrometry (MS) data. Additionally, for determining the stereochemical structure of compound **1**, it was subjected to a chemical reaction.

**Figure 2:**
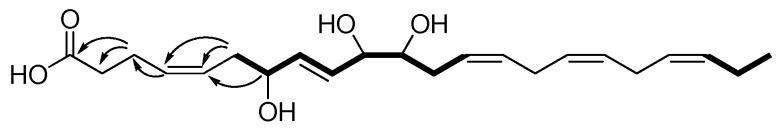
Key correlated spectroscopy (COSY) (bold lines) and heteronuclear multiple-bond correlation spectroscopy (HMBC) (arrows) correlations of compound **1**.

Compound **1** was isolated in the form of an amorphous gum. Based on the negative ion peak of [M − H]^−^ at *m*/*z* = 377.2325 (Δ −0.3 mmu) from the high resolution ElectroSpray Ionization (ESI) mass spectrum, the molecular formula of **1** was determined to be C_22_H_34_O_5_, indicating six degrees of unsaturation. The infrared (IR) spectrum showed absorption bands corresponding to the hydroxyl (3329 cm^−1^) and carbonyl (1716 cm^−1^) groups. The integration of ^1^H NMR and edited Heteronuclear Single Quantum Correlation (HSQC) spectra of compound **1** revealed seven methylenes, three oxymethines, and one methyl group. Apart from this, one carbonyl carbon was also detected from the heteronuclear multiple-bond correlation spectroscopy (HMBC) spectrum. The molecular structure was elucidated by interpreting the 1D and 2D NMR spectra (Table 1). First, the two partial structures shown in bold lines in Figure 2 were constructed using the Correlation spectroscopy (COSY). One terminal (C-6) in the hydroxylated chain unit was connected with an allyl group from the HMBC correlations of H-7/C-5, H-6/C-4, H-6/C-5, and H-4/C-3. The other HMBC correlations of H-3/C-2 and H-3/C-1 showed a connection between the allylic carbon (C-3) and one methylene, and the termination with a carboxylic acid. On the other hand, the chemical shifts of the remaining four olefinic and two methylene signals suggested a linear structure with methylene-mediated double bonds. This unit might be placed in between the two partial structures, which was confirmed by the Total Correlation Spectroscopy (TOCSY) correlations with the methylene protons at 2.81 and 2.84 ppm. The Δ^4,5^, Δ^13,14^, Δ^16,17^, and Δ^19,20^ were found to be *cis* forms, based on the chemical shifts of their allylic carbons [17]. In contrast, Δ^8,9^ was found to be *trans* form, based on the large coupling constant (^3^*J*_HH_ = 15.7 Hz) of the olefinic protons. Accordingly, compound **1** was determined to be a new trioxilin, named as (4*Z*,8*E*,13*Z*,16*Z*,19*Z*)-7,10,11-trihydroxydocosapentaenoic acid. This structure was verified by the fragment ion peaks in the negative ESI-MS/MS spectrum as shown in Figure S1.

**Figure 3:**
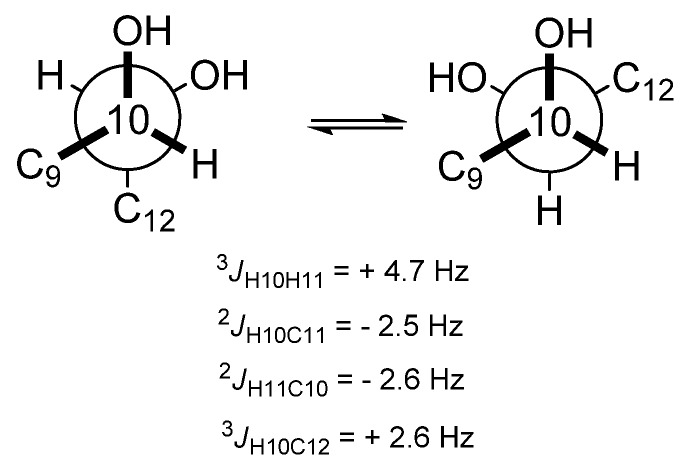
Conformers of C-10 and C-11 of compound **1**, based on *J*-based configuration analysis.

The stereochemistry of the three chiral centers in **1** was determined using a *J*-based configuration analysis (JBCA) [18] and the modified Mosher’s method. For the *J*-based analysis, the homo- and heteronuclear coupling constants were measured from the well-split proton signals and Heteronulear Long-range Coupling (HETLOC) spectrum, respectively. The intermediate homonuclear coupling constant (*J*_HH_ = 4.7 Hz) between H-10 and H-11 indicated the interconversion of *trans* and *gauche* conformations of the two protons. Moreover, the two heteronuclear coupling constants of H-10/C-11 and H-11/C-10 are commonly considered as intermediate values, rationalized by the interconversion of the two conformers (Figure 3). The intermediate homo- and two-bond heteronuclear coupling constants resulted in a *syn* relationship between C-10 and C-11. A small heteronuclear coupling constant of H-10/C-12 supported the relationship between C-10 and C-11. In addition, for determination of the absolute stereochemistry of C-7, C-10, and C-11 in compound **1**, Mosher’s analysis was performed, in which the compound was treated with *R-*(−)- and *S-*(+)-α-Methoxy-α-trifluoromethylphenylacetyl (MTPA) chlorides after methylation with trimethylsilyl-diazomethane. Each product was esterified with three secondary alcohols in compound **1**, which was recognized by the molecular fragment with *m*/*z* = 1041 in the low resolution ESI-MS spectrum. The protons near the three chiral centers of the *S/R*-MTPA esters were assigned with the aid of the COSY and TOCSY spectra, and then the differences in chemical shifts of the corresponding protons were then calculated (Figure S3). The absolute configurations of both C-10 and C-11 were determined to be *S*, based on the interpretation of the 1,2-diol system by Riguera et al. [19]. Finally, Mosher’s analysis of C-7 also indicated *S* configuration. Compound **1** was thus determined to be (4*Z*,8*E*,13*Z*,16*Z*,19*Z*)-7(*S*),10(*S*),11(*S*)-trihydroxydocosapentaenoic acid—a metabolite biosynthesized through hepoxilin, which was derived from DHA by 11-lipoxygenase.

Compound **2** was isolated as a colorless gum from the same extract. The exact mass of [M − H]^−^ was measured at *m/z* = 865.5135, and molecular formula was found to be C_47_H_78_O_12_S (theoretical *m/z* = 865.5136). The ^1^H NMR spectrum for compound **2** displayed intense aliphatic and olefinic proton signals corresponding to long saturated carbons and polyunsaturated carbons, respectively; it also showed two triplet-methyl protons, which indicated two terminal groups. The ^13^C NMR spectrum indicated two acyl groups with long carbon chains from two carbonyl carbons, and crowded carbon signals in the ranges of 30–31 ppm and 128–130 ppm. In addition, the presence of a SO_3_H functional group was inferred from the absorption bands at 1168 and 1034 cm^−1^ in the IR spectrum, and from the molecular formula and the MS/MS with a loss of 81 amu. Based on this information, the COSY correlations and the proton coupling constants indicated a sulfoquinovose unit. The coupling constant for the anomeric proton was measured to be 3.9 Hz, and was therefore assigned α form. This was consistent with previous reports of the proton chemical shifts and the coupling constants of 6-sulfo-α-d-quinovopyransyl group [20]. Additional HMBC correlations with the two acyl groups suggested the structure of a SQDG. Furthermore, the two fatty acyl groups were determined by MS/MS fragment ions at *m*/*z* = 537 and 609 as a docosahexanenoyl group and a hexdecanoyl group, respectively (Figure S4). After hydrolysis of compound **2**, a molecular ion peak of [M − H]^−^ at *m*/*z* = 255 in the LRESI-MS spectrum confirmed the presence of hexadecanoic acid. The NMR spectral data for protons and carbons in the two acyl groups were assigned using the two dimensional (2D) NMR spectra (Table 2). Specifically, the assignments of H-2′–H-4′ and H-2′′–H-4′′ were apparently corroborated by the TOCSY correlations of H-2′ and H-2″ which had HMBC correlations with C-1′ and C-1″, respectively. Based on the two acyl groups and the sulfoquinovose, the linkage of the sulfoquinovosyl and the hexadecanoyl moieties to the S_N_1 and S_N_3 positions was recognized from the HMBC correlations of H-3/C-1′′′ and H-1/C-1′, respectively. Therefore, the structure of compound **2** was determined to be 1-*O*-hexadecanosy-2-*O*-docosahexaenoyl-3-*O*-(6-sulfo-α-d-quinovopyranosyl)-glycerol. The configuration of the S_N_2 center was determined to be *S* form by comparison of the optical rotation of compound **2** with that reported for other similar compounds [21,22]. Compound **2** was found to be a typical SQDG compound, but had a high unsaturated fatty acid (DHA) and a saturated fatty acid.

Compounds **1** and **2** showed anti-inflammatory effects on LPS-activated RAW264.7 macrophage cells. The amount of nitrite released into culture media increased five-fold after exposure to 100 ng·mL^−1^ of LPS for 24 h. The NO production induced by LPS was significantly suppressed by pretreatment with **1** and **2**, without affecting cell viability; the IC_50_ values of compounds **1** and **2** were calculated to be 22.30 and 10.76 μM, respectively.

The in vitro cytotoxic activity of compounds **1** and **2** against hepatocarcinoma (HepG2), neuroblastoma (Neuro-2a), and colon cancer (HCT-116) cells was also measured. Compounds **1** and **2** did not show any cytotoxicity against any of the three cell lines with a concentration range of 0.1–20 μM. Weak cytotoxicity was observed for compound **1** against Neuro-2a neuroblastoma cells; the relative cell viability was found to be 68%–72% at a concentration range of 0.1–20 μM.

The two new isolated compounds were derived from DHA, which is abundant in the dinoflagellates *O. marina*, and is important for human health. To the best of our knowledge, compound **1**—a trioxilin—was first isolated from *O. marina*, and compound **2** is the first SQDG with a DHA ester. The two compounds exhibited strong NO-inhibitory activity on RAW264.7 cells. Although dinoflagellates are best known for the toxin producers, they have provided novel structures with biological activity (e.g., anticancer) [16,23]. Furthermore, microalgae including dinoflagellates have the ability to produce diverse metabolites through changes in culture conditions, and might be a promising source for drug discovery.

## 3. Materials and Methods

### 3.1. General

Optical rotations were measured on a JASCO P-1010 digital polarimeter (Tokyo, Japan) with a 5 cm cell. IR spectra were recorded on a JASCO FT/IR 4100 spectrometer. The 1D and 2D NMR spectra were obtained using a Varian VNMRS 500 spectrometer (Palo Alto, CA, USA) in a CD_3_OD solvent and CDCl_3_. High-resolution ESI mass spectra were acquired using a Waters SYNAPT G2, courtesy of Korea Basic Science Institute (Ochang Center, Korea). The ESI-MS/MS spectra were obtained in the enhanced product ion scan mode on an ABSCIEX QTRAP 3200 (Foster, CA, USA).

### 3.2. Material

*O. marina* was isolated from Gunsan, Korea in May 2001, when the water temperature and salinity were 16 °C and 27.7 psu, respectively. Clonal cultures of *O. marina* were fed on dried yeasts (*Saccharomyces cerevisiae*, Red Star, Lesaffre Yeast Corporation, Milwaukee, MI, USA) at 22–24 °C under 10 μE·m^−2^·s^−1^ of continuous cool white fluorescent light. The yeast (0.1 g·L^−^^1^) was sterilized by autoclave at 121 °C for 30 min and then supplied to the predator cells daily. No yeast was supplied on the last day to ensure the complete elimination of yeasts from the culture waters.

As the concentration of *O. marina* increased, the culture was transferred to 2 L and 20 L polycarbonate bottles, then to a 200 L tank, and finally to a 1000 L tank. *O. marina* cells were harvested by centrifugation (15,000 rpm, 10 h) when cell concentration reached around 70,000–90,000 cells·mL^−1^. The harvested cells were stored in a deep freezer (−75 °C); the moisture was removed using a freeze-dryer (−55 °C, 5 mTorr, 24 h).

### 3.3. Extraction and Isolation

The freeze-dried cells were extracted with a 100% MeOH solvent to yield 16 g of the extract. The extract was first partitioned into methylene chloride and H_2_O, and the organic layer was then repartitioned into 85% MeOH and hexane to eliminate non-polar fatty acids. The aqueous layer was subjected to HPLC with a size exclusion column (100 Å, 300 × 7.8 mm) by eluting the 100% MeOH solvent to generate 10 fractions (Frs 1–10). Fr 6 (35 mg), which was collected at the retention time of 20 min, was purified using reversed-phase HPLC (Synergi polar-RP, 250 mm × 4.6 mm) by gradient elution by changing H_2_O:MeOH (*v*/*v*) from 60:40 to 100:0 in 35 min, and compound **1** (10 mg) was obtained. Frs 7–8 (55 mg) were combined and then purified with the same protocol described above to obtain compound **2** (13 mg).

Compound **1**: ${\left[\alpha \right]}_{25}^{D}$ +4.7 (*c* 1.0, MeOH); IR (film) ν_max_ (cm^−1^): 3330, 2900, 1716, 1401; ^1^H and ^13^C NMR data are given in Table 1; HRESI–MS *m*/*z* = 377.2325 for [M − H]^−^ (calcd *m*/*z* = 377.2328 for C_22_H_33_O_5_); MS/MS *m*/*z* = 112 for [C_6_H_8_O_2_]^−^, *m*/*z* = 137 for [C_8_H_11_O_3_]^−^ − H_2_O, and *m*/*z* = 181 for [C_10_H_15_O_4_]^−^ − H_2_O.

Compound **2**: ${\left[\alpha \right]}_{25}^{D}$ +34.8 (*c* 0.1, MeOH); IR (film) ν_max_ (cm^−1^): 3447, 2923, 1736, 1169, 1034; ^1^H and ^13^C NMR data are given in Table 2; HRESI–MS *m*/*z* = 865.5135 for [M − H]^−^ (calcd *m*/*z* = 865.5136 for C_47_H_77_O_12_S); MS/MS *m*/*z* = 81 for [SO_3_]^−^, *m*/*z* = 255 for [C_6_H_1__0_O_7_S]^−^, *m*/*z* = 537 for [C_25_H_46_O_11_S]^−^ − H_2_O, and *m*/*z* = 609 for [C_31_H_46_O_11_S]^−^ − H_2_O.

### 3.4. MTPA Reaction of Reduced Compound ***1***

Compound **1** was first converted into its methyl ester by reaction with diazomethane in MeOH. After stirring the solution of reduced compound **1** (0.3 mg) and 4-dimethylamino pyridine (30 μL) in CDCl_3_ (0.1 mL) at 30 °C, *R*-(−)–MTPA-Cl (5 μL) was added. The reaction progress was monitored by thin-layer chromatography (TLC) on silica gel 4:1 Hex/EtOAc. After ~24 h, the reaction was quenched by the addition of H_2_O and dimethyl ether. The organic layer was then concentrated in vacuo. The crude product mixture was eluted with silica-gel Solid Phase Extraction (SPE) using hexane/ethyl acetate (5:1) to produce the *S*-MTPA-ester reduced **1a** as a pale-yellow gum. ^1^H NMR (CDCl_3_, 500 MHz) 5.785 (H-8), 5.774 (H-9), 4.180 (H-7), 4.136 (H-10), 3.669 (H-11), 2.300 (H-6), 2.293 (H-12), 2.168 (H-12); LRESI-MS *m*/*z* = 1041 for [M + H]^+^.

In an entirely analogous way, the *R*-MTPA-ester reduced **1b** was also produced using *S*-MTPA-Cl. ^1^H NMR (CDCl_3_, 500 MHz) 5.783 (H-8) 5.773 (H-9), 4.179 (H-7), 4.126 (H-10), 3.658 (H-11), 2.315 (H-6), 2.286 (H-12), 2.163 (H-12); LRESI-MS *m*/*z* = 1041 for [M + H]^+^.

### 3.5. Cell Cultures

RAW264.7 (mouse macrophage cells), HCT-116 (human colon cancer cells), and HepG2 (human liver carcinoma cells) were obtained from the Korea Cell Line Bank (Seoul, Korea). Neuro-2a (mouse brain neuroblastoma cells) were purchased from ATCC (Manassas, VA, USA). Cells were maintained in DMEM containing 10% FBS with penicillin (100 IU·mL^−1^) and streptomycin (10 mg·mL^−1^) at 37 °C in a 5% CO_2_ atmosphere and 95% relative humidity.

### 3.6. Estimation of NO Production

RAW264.7 macrophage cells were plated in 48-well plates (1 × 10^5^ cells·mL^−1^, 300 μL per well) for 18 h. To remove all the traces of phenol red, the cell culture was washed and the medium was replaced with fresh medium without phenol red. Cells were treated with the sample to be tested for 1 h before exposure to 100 ng·mL^−1^ of LPS. After 24 h of incubation, the nitrite content in the culture media was measured using the Griess reagent (1% sulfanilamide, 0.1% naphthylethylenediamine dihydrochloride, and 2% phosphoric acid). The sample aliquots (100 μL) were mixed with 100 μL of the Griess reagent in a 96-well plate and incubated at room temperature for 10 min. The absorbance at 550 nm was then measured on a microplate reader, and the concentrations were determined using a nitrite standard curve [24].

### 3.7. Estimation of Cytotoxicity

Compounds to be tested were dissolved in dimethyl sulfoxide (DMSO) (final concentration of 0.1%) and diluted in serum-free culture medium. Before the assay, the cells were seeded and incubated for 24 h in 48-well plates (HCT-116: 1 × 10^5^ cells·mL^−1^; neuro-2a cells: 2 × 10^5^ cells·mL^−1^; HepG2 cell: 5 × 10^4^ cells·mL^−1^; 100 μL per well). The cells were then treated with the vehicle or the compounds at concentrations indicated for 48 h. The inhibitory activity of each compound on cell proliferation was assessed by the 3-(4,5-dimethylthiazol-2-yl)-2,5-diphenyltetrazolium bromide (MTT) assay. Cells were incubated with 2 mg·mL^−1^ of MTT for 2 h. The supernatant was then aspirated, and 200 μL of DMSO was added to dissolve the formazan. After all the crystals were completely dissolved, the absorbance at 450 nm was measured on a microplate reader. The data were expressed as percentage of viable cells relative to vehicle-treated control cultures. The data were expressed as the means of three independent experiments. IC_50_ values refer to concentration at which 50% inhibition was achieved, and were calculated from regression lines with at least five different concentrations.

## 4. Conclusions

Two types of compounds—a trioxilin and a SQDG—were isolated from a massive culture of the heterotrophic dinoflagellate *O. marina*. The complete structures of these compounds were determined using NMR spectroscopy and chemical reactions. The trioxilin was found to be derived from DHA, and the polar lipid SQDG was found to contain a docosahexaenoyl substituent. Compounds **1** and **2** significantly suppressed the NO production induced by LPS on RAW264.7 cells without affecting cell viability.

## Figures and Tables

**Table 1:** Nuclear Magnetic Resonance (NMR) spectral data for compound **1** in CD_3_OD.

Position	δ_C_	δ_H_ (*J* in Hz)
1	177.3, C	
2	35.2, CH_2_	2.31, m
3	24.2, CH_2_	2.33, m
4	131.1, CH	5.46, m
5	127.6, CH	5.47, m
6	36.3, CH_2_	2.31, m
7	73.0, CH	4.10, dt (6.1, 6.1)
8	136.2, CH	5.72, dd (15.7, 6.1)
9	130.9, CH	5.78, dd (15.7, 6.1)
10	76.0, CH	3.96, dd (6.1, 4.7)
11	75.9, CH	3.53, dt (8.3, 4.7)
12	31.8, CH_2_	2.18, dd (14.9, 8.3); 2.33, m
13	127.4, CH	5.49, m
14	130.7, CH	5.42, m
15	26.8, CH_2_	2.84, dd (6.6, 6.1)
16	129.0, CH	5.34, m
17	129.5, CH	5.34, m
18	26.4, CH_2_	2.81, dd (7.1, 5.9)
19	128.2, CH	5.29, m
20	132.8, CH	5.37, m
21	21.5, CH_2_	2.08, q (7.3)
22	14.7, CH_3_	0.96, t (7.6)

**Table 2:** NMR spectral data for compound **2** in CD_3_OD.

Position	δ_C_	δ_H_ (*J* in Hz)
1	64.2, CH_2_	4.18, dd (11.0, 6.9); 4.49, dd (11.0, 2.9)
2	71.9, CH	5.31, m
3	67.1, CH_2_	3.57, dd (10.8, 6.4); 4.11, dd (10.8, 5.1)
1′	175.1, C	
2′	35.0, CH_2_	2.31, t (7.6)
3′	26.0, CH_2_	1.54, q (7.6)
4′	30.2, CH_2_	1.30, m
5′–13′	30.8–30.5, CH_2_	1.30, m
14′	33.1, CH_2_	1.26, m
15′	23.7, CH_2_	1.30, m
16′	14.4, CH_3_	0.89, t (6.9)
1″	174.2, C	
2″	35.1, CH_2_	2.39, m
3″	23.7, CH_2_	2.39, m
4″	130.1, CH	5.37, m
5″, 7″–8″,	129.5–129.0, CH_2_	5.36, m
10″–11″, 13″–14″		
16″–17″, 19″		
6″, 9″ 12″, 15″, 18″	26.6–26.5, CH_2_	2.85, m
20″	132.8, CH	5.36, m
21″	21.5, CH_2_	2.08, q (7.6)
22″	14.7, CH_3_	0.96, t (7.6)
1‴	100.1, CH	4.75, d (3.9)
2‴	73.5, CH	3.39, dd (9.8, 3.9)
3‴	74.9, CH	3.62, t (9.8)
4‴	75.1, CH	3.08, dd (9.8, 9.1)
5‴	69.9, CH	4.07, td (9.1, 2.0)
6‴	54.3, CH_2_	2.91, dd (14.2, 9.1); 3.34, dd (14.2, 2.0)

## Supplementary Materials

The following are available online at www.mdpi.com/1660-3397/15/3/57/s1, Figure S1: MS/MS fragmentation of compound **1** and its MS/MS spectrum, Figure S2: Conformers of C-10 and C-11 of compound **1**, based on *J*- based configuration analysis, Figure S3: Differences in chemical shifts of key protons of *S*-MTPA ester(**1a**) and *R*-MTPA ester(**1b**), Figures S4–S9: 1D and 2D NMR spectra of compound **1**, Figure S10: MS/MS fragmentation of compound **2**, Figures S11–S17: 1D and 2D NMR spectra of compound **2**.
