# Supplementary material for: Characterization of a New Trioxilin and a Sulfoquinovosyl Diacylglycerol with Anti-Inflammatory Properties from the Dinoflagellate Oxyrrhis marina
Source: Mar Drugs. 2017 Feb 27;15(3):57. doi: 10.3390/md15030057 (PMC5367014; doi:10.3390/md15030057)
Supplement: Supplementary file 1 [file marinedrugs-15-00057-s001.pdf]

Supplementary Materials

Characterization of a new trioxilin and a sulfoquinovosyl diacylglycerol with anti-inflammatory properties from the dinoflagellate *Oxyrrhis marina*

Eun Young Yoon, A Reum Yang, Jaeyeon Park, Seung Joo Moon, Eun Ju Jeong and Jung-Rae Rho

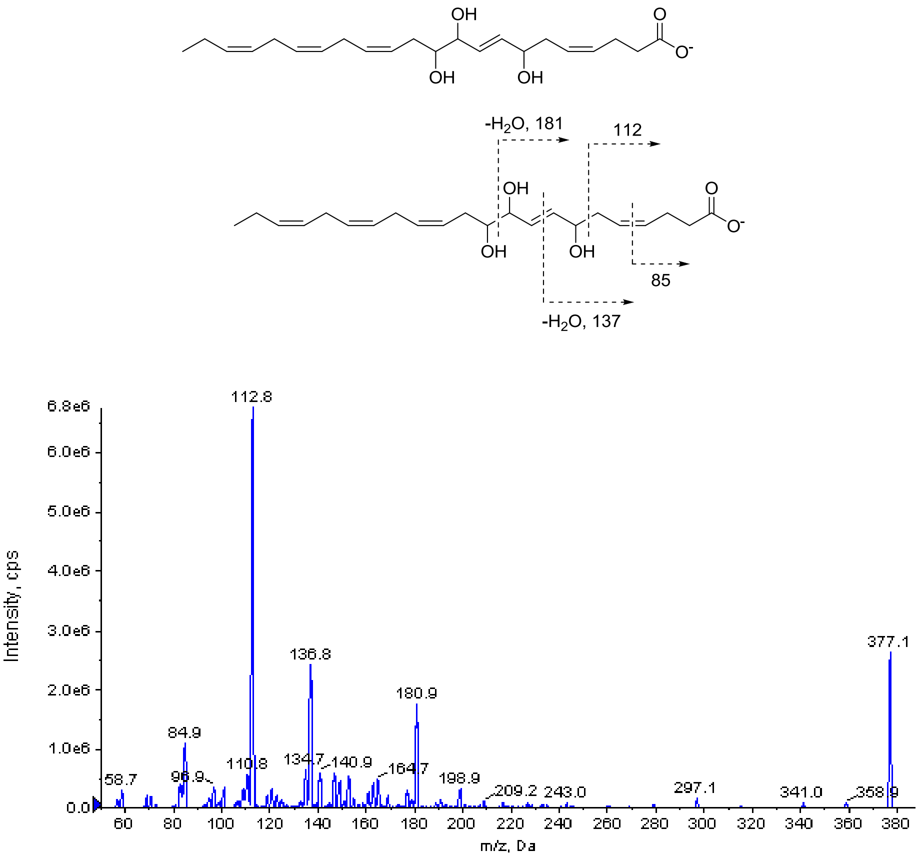

Figure S1. MS/MS fragmentation of compound **1**.

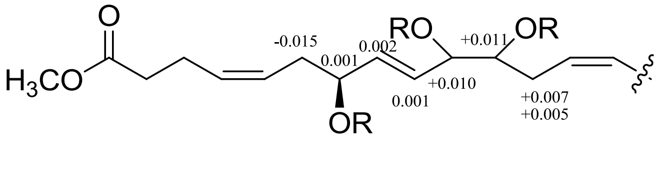

1a R = *S*-MTPA
1b R = *R*-MTPA

Figure S2. Differences in chemical shifts of key protons of *S*-MTPA ester (**1a**) and *R*-MTPA ester (**1b**).

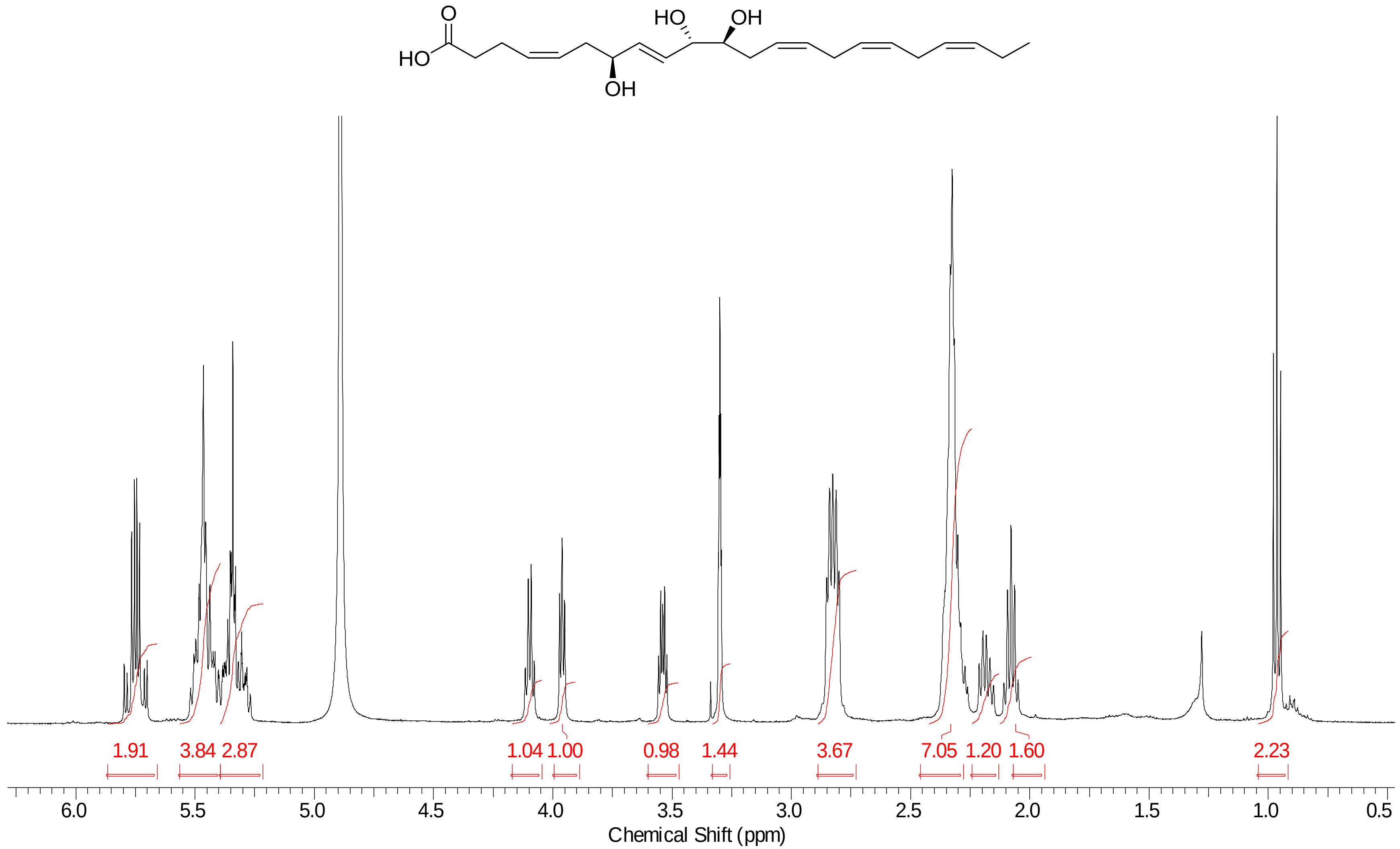

Figure S3. ^{1}H NMR spectrum of compound **1** at 500 MHz.

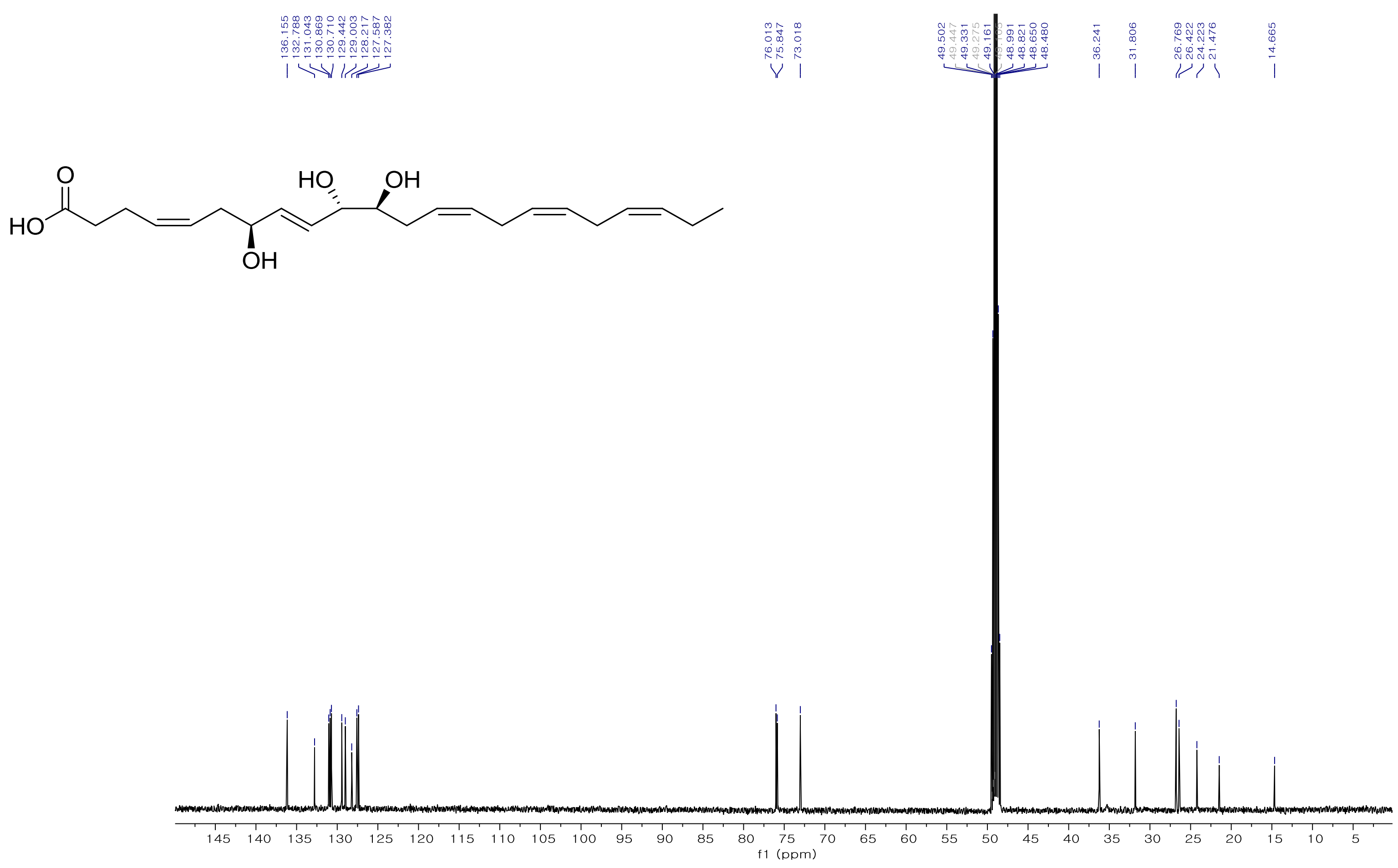

Figure S4. ^{13}C NMR spectrum of compound **1** at 125 MHz.

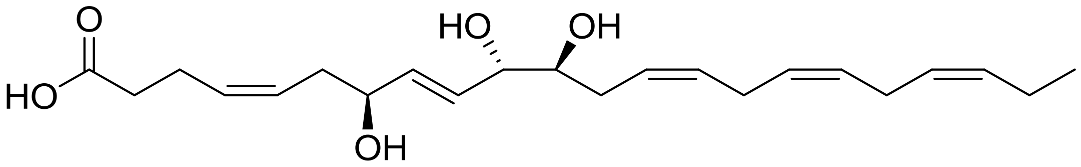

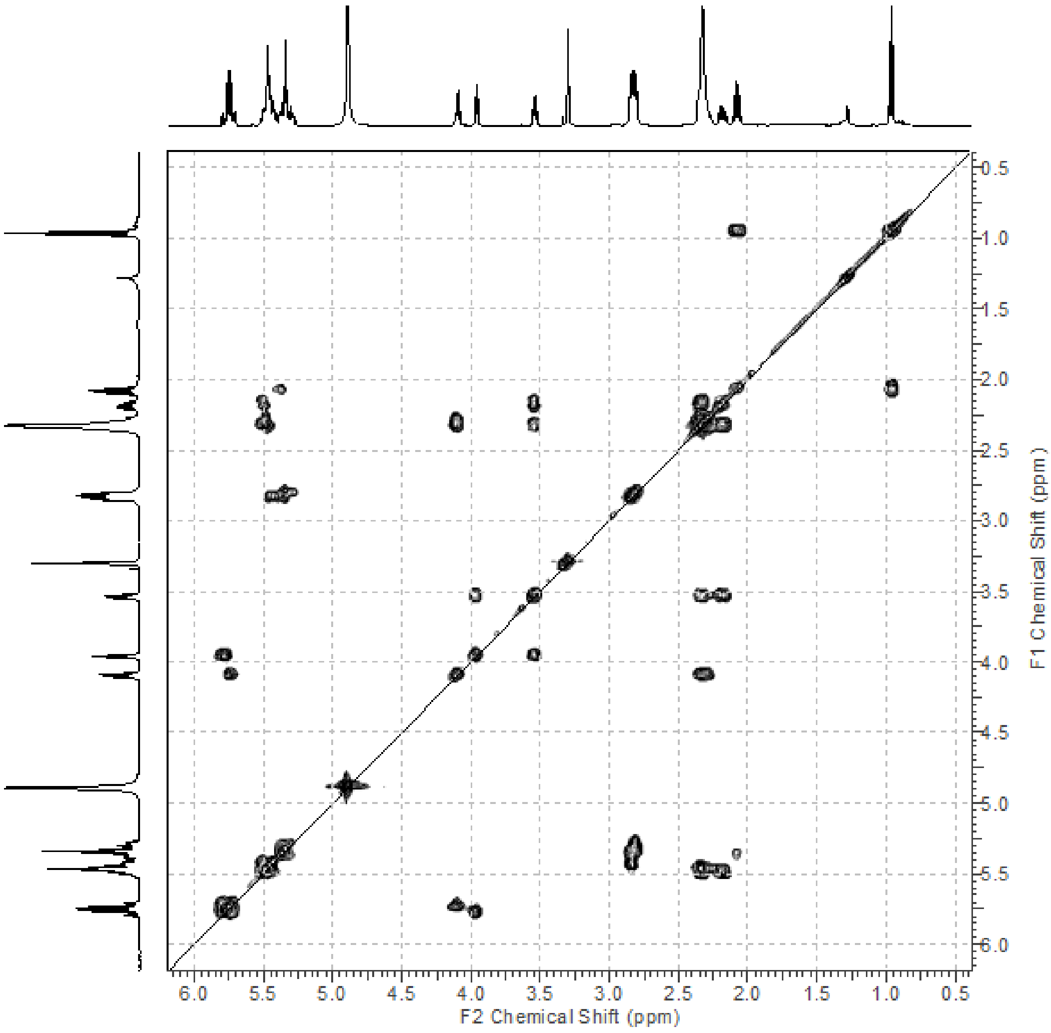

Figure S5. COSY NMR spectrum of compound **1** at 500 MHz.

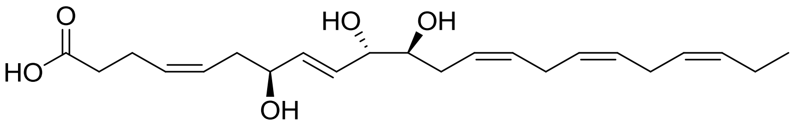

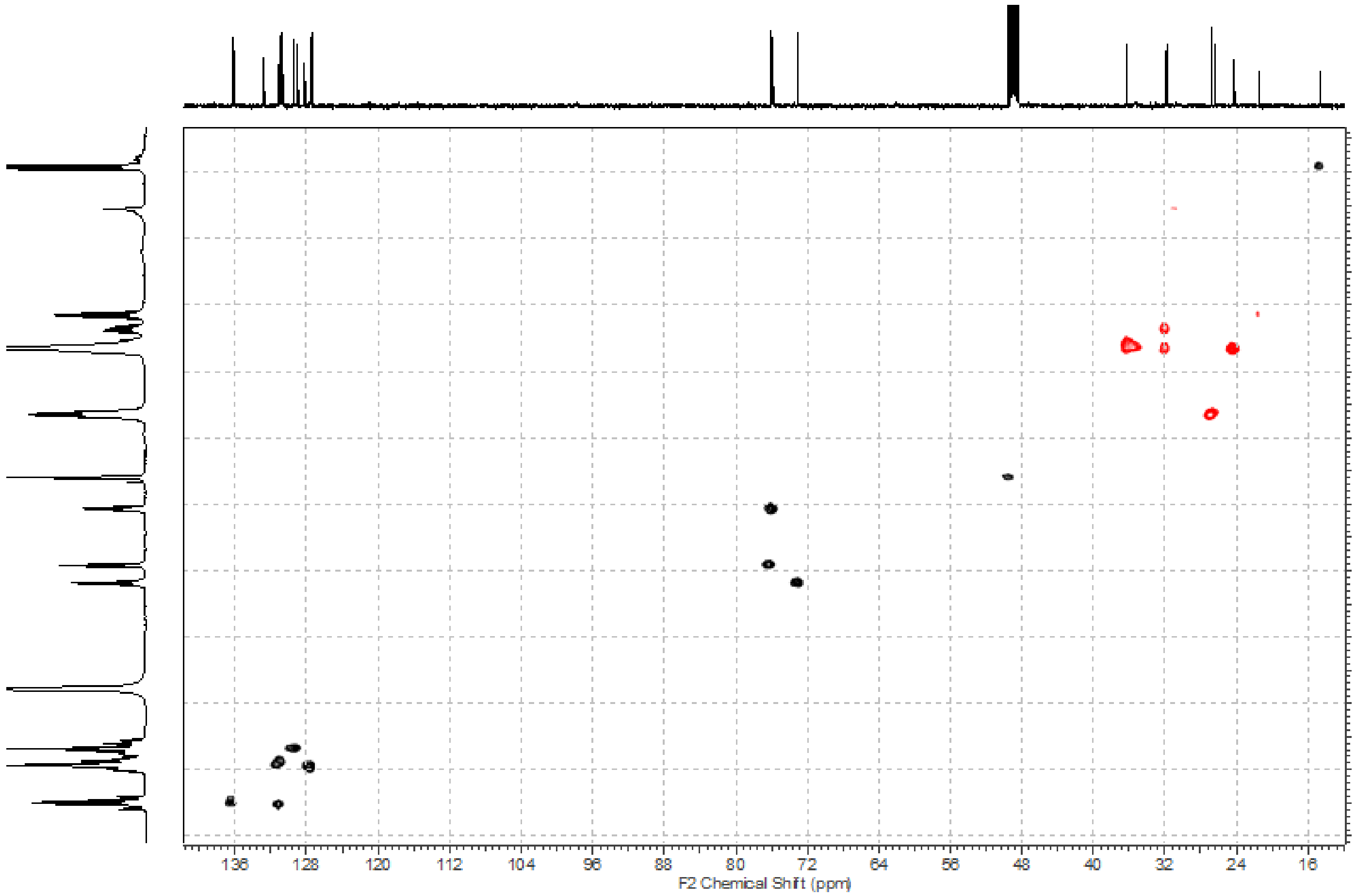

Figure S6. HSQC NMR spectrum of compound **1** at 500 MHz. (Black: CH, CH_3; Red: CH_2)

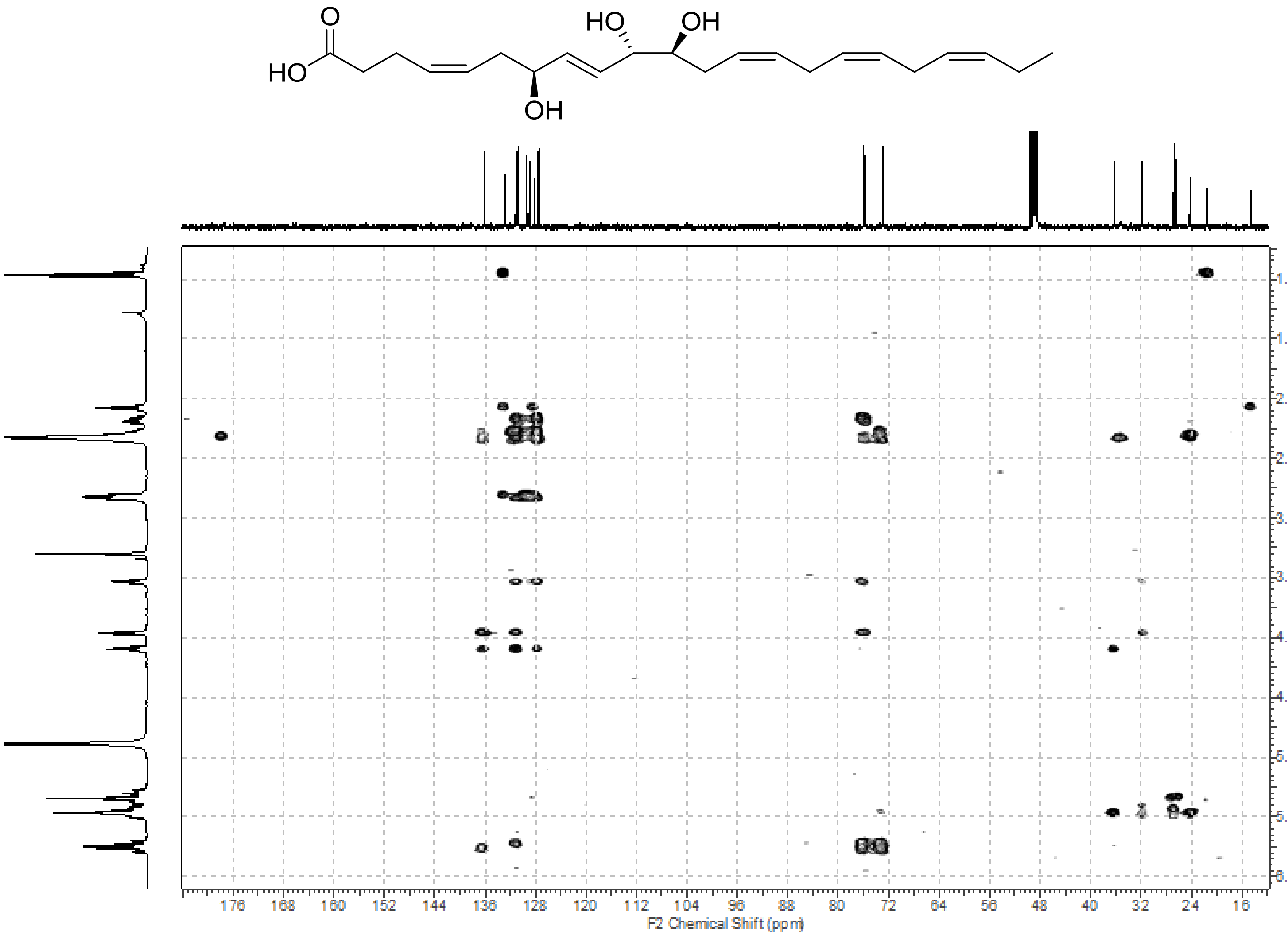

Figure S7. HMBC NMR spectrum of compound **1** at 500 MHz.

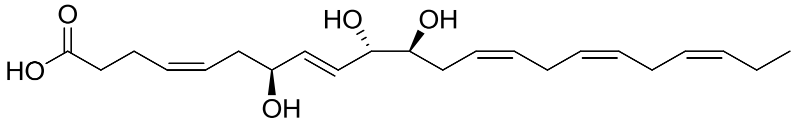

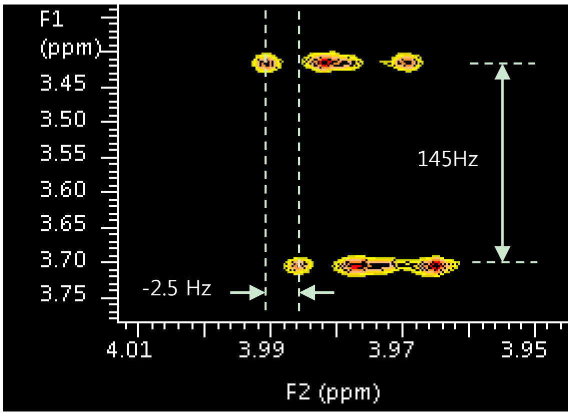

$^{2}J_{H_{10}C_{11}} = -2.5$ Hz

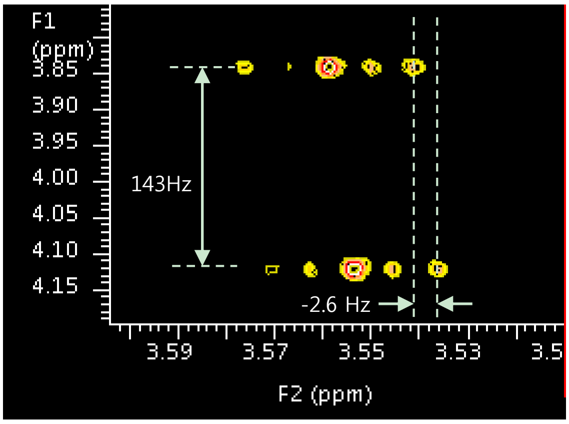

$^{2}J_{H_{11}C_{10}} = -2.6$ Hz

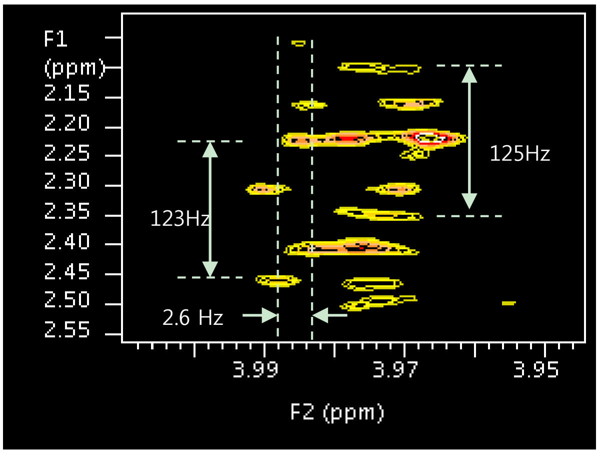

$^{3}J_{H_{10}C_{12}} = +2.6$ Hz

Figure S8. Key HETLOC NMR cross peaks of compound **1** at 500 MHz.

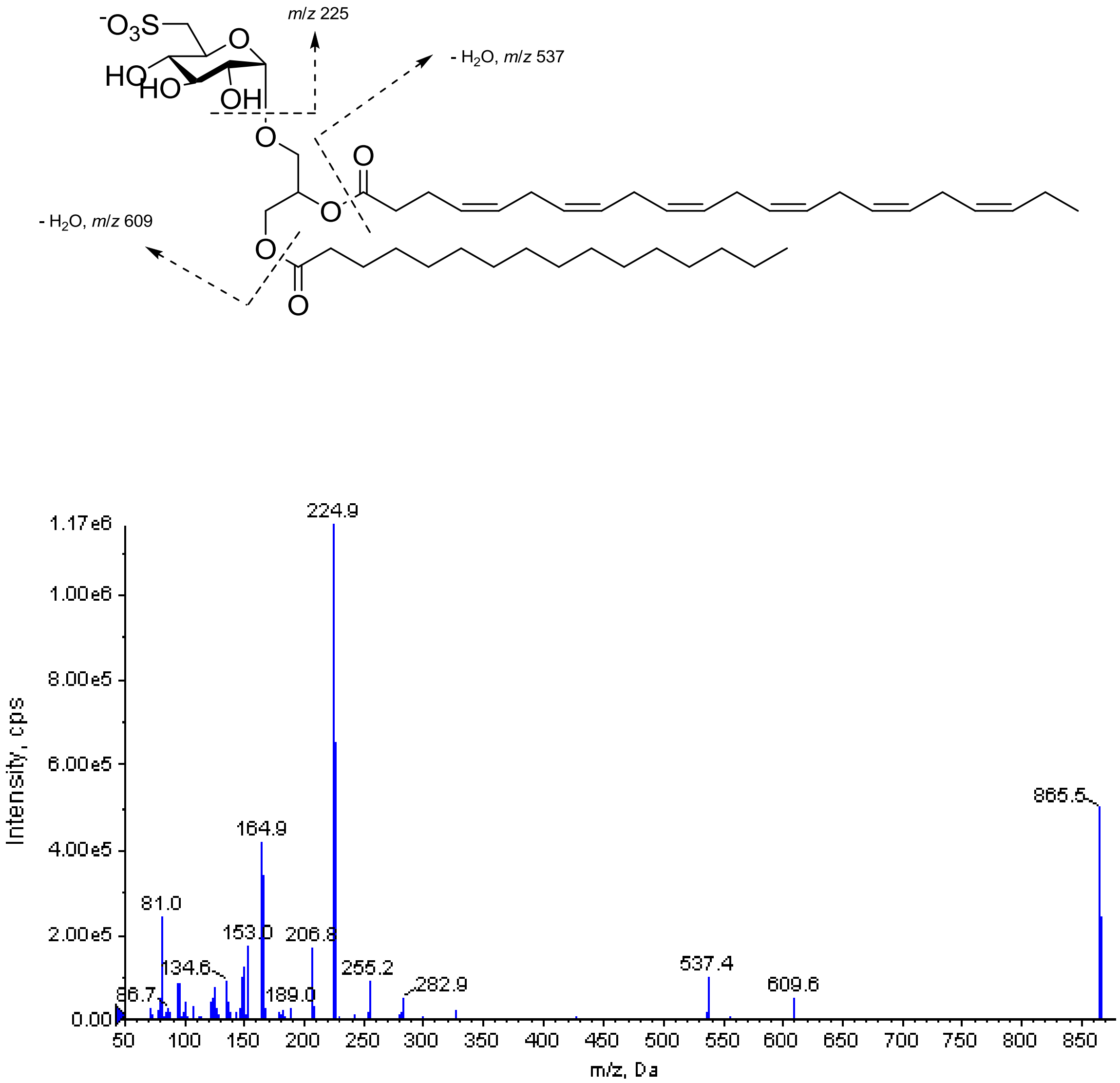

Figure S9. MS/MS fragmentation of compound **2**.

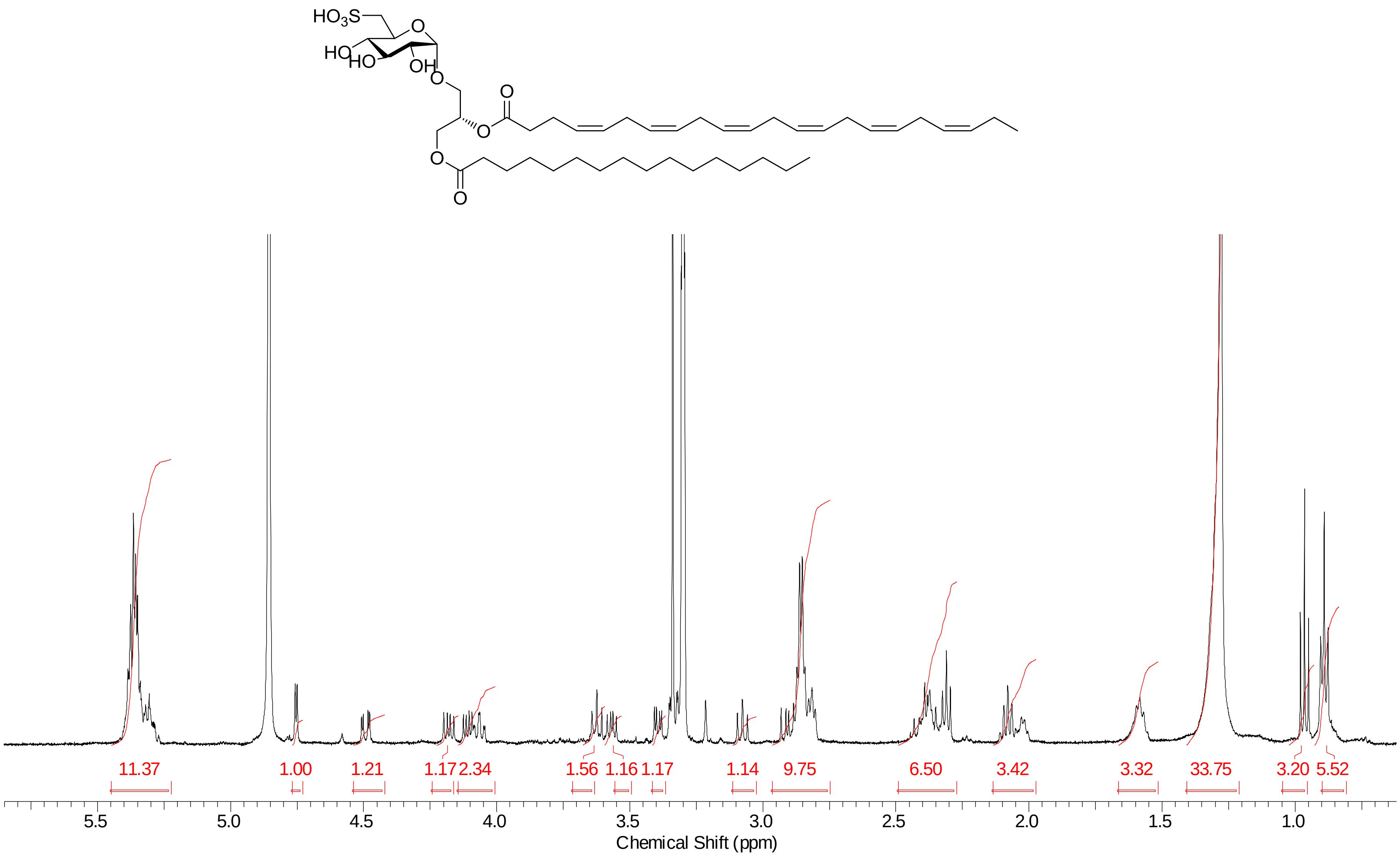

Figure S10. ^{1}H NMR spectrum of compound **2** at 500 MHz.

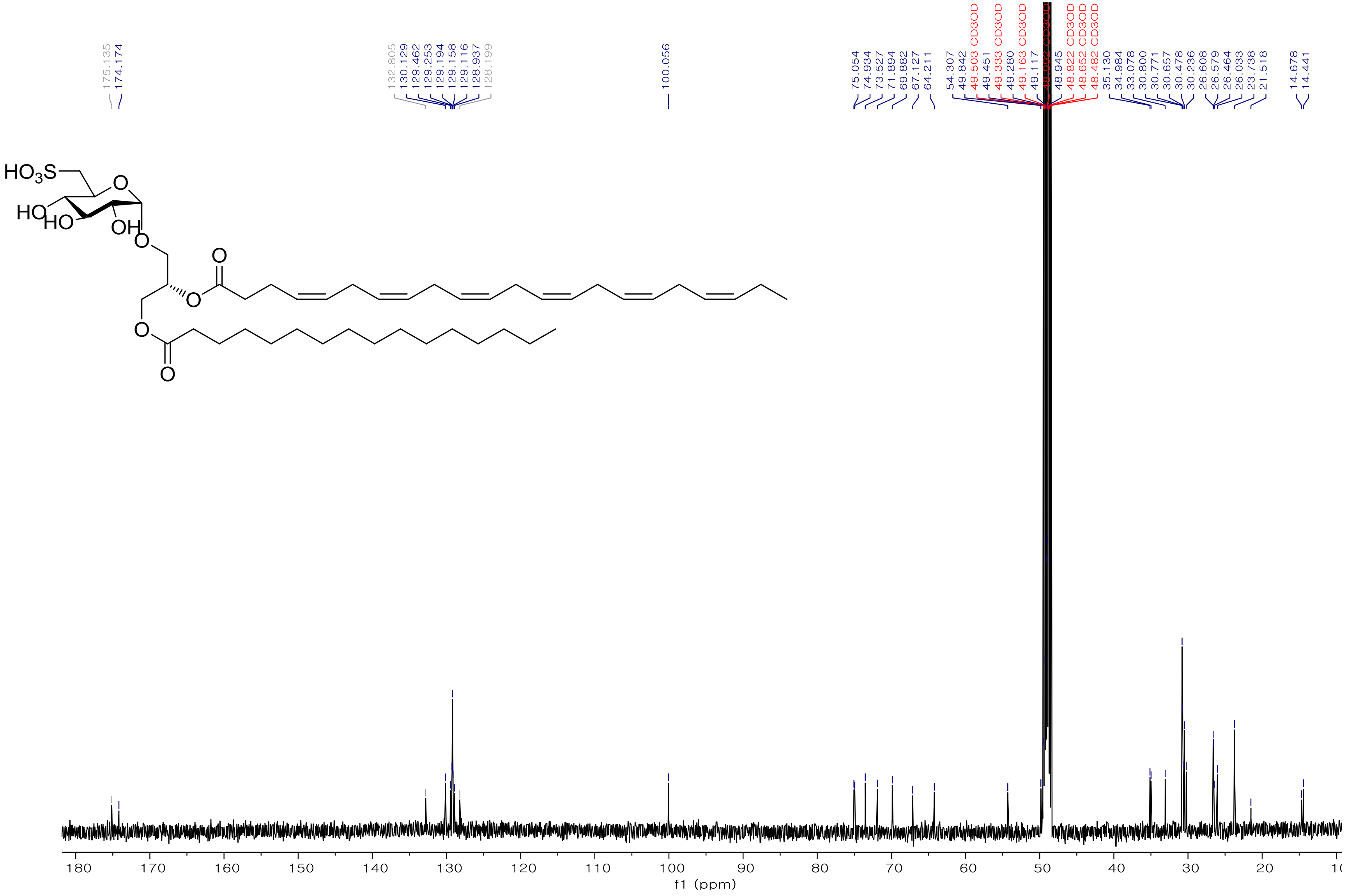

Figure S11. ^{13}C NMR spectrum of compound **2** at 125 MHz.

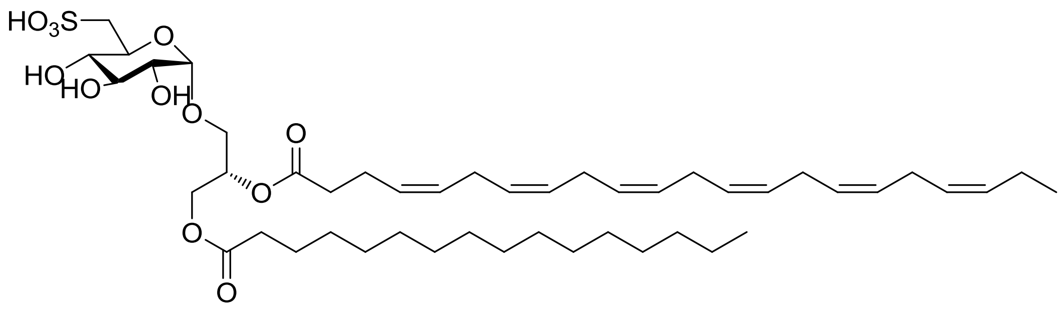

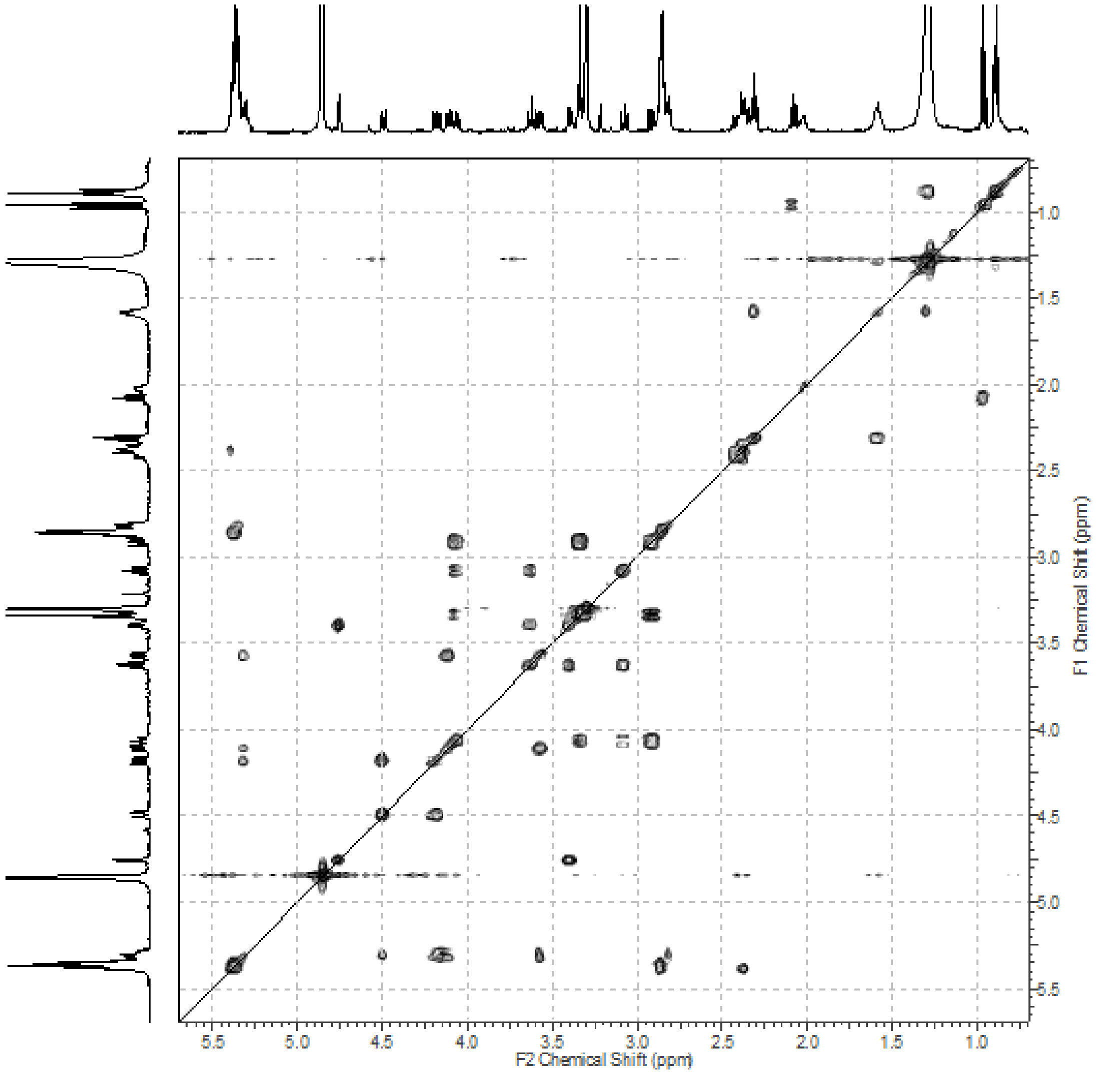

Figure S12. COSY NMR spectrum of compound **2** at 500 MHz.

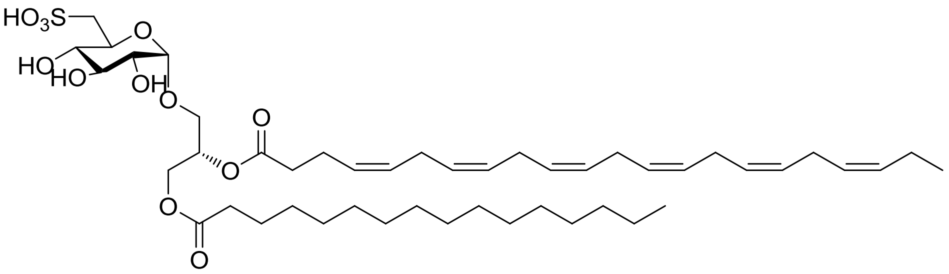

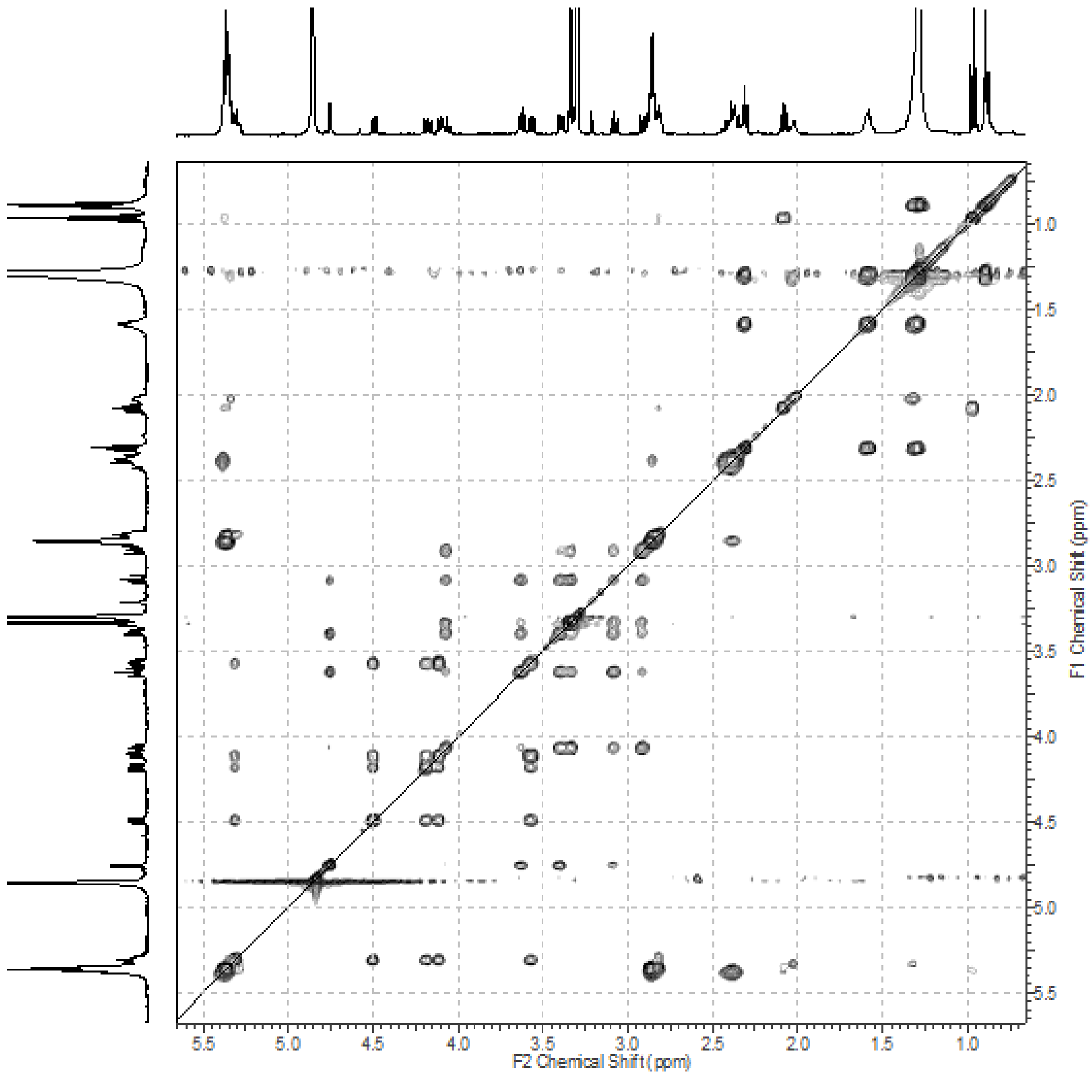

Figure S13. TOCSY NMR spectrum of compound **2** at 500 MHz.

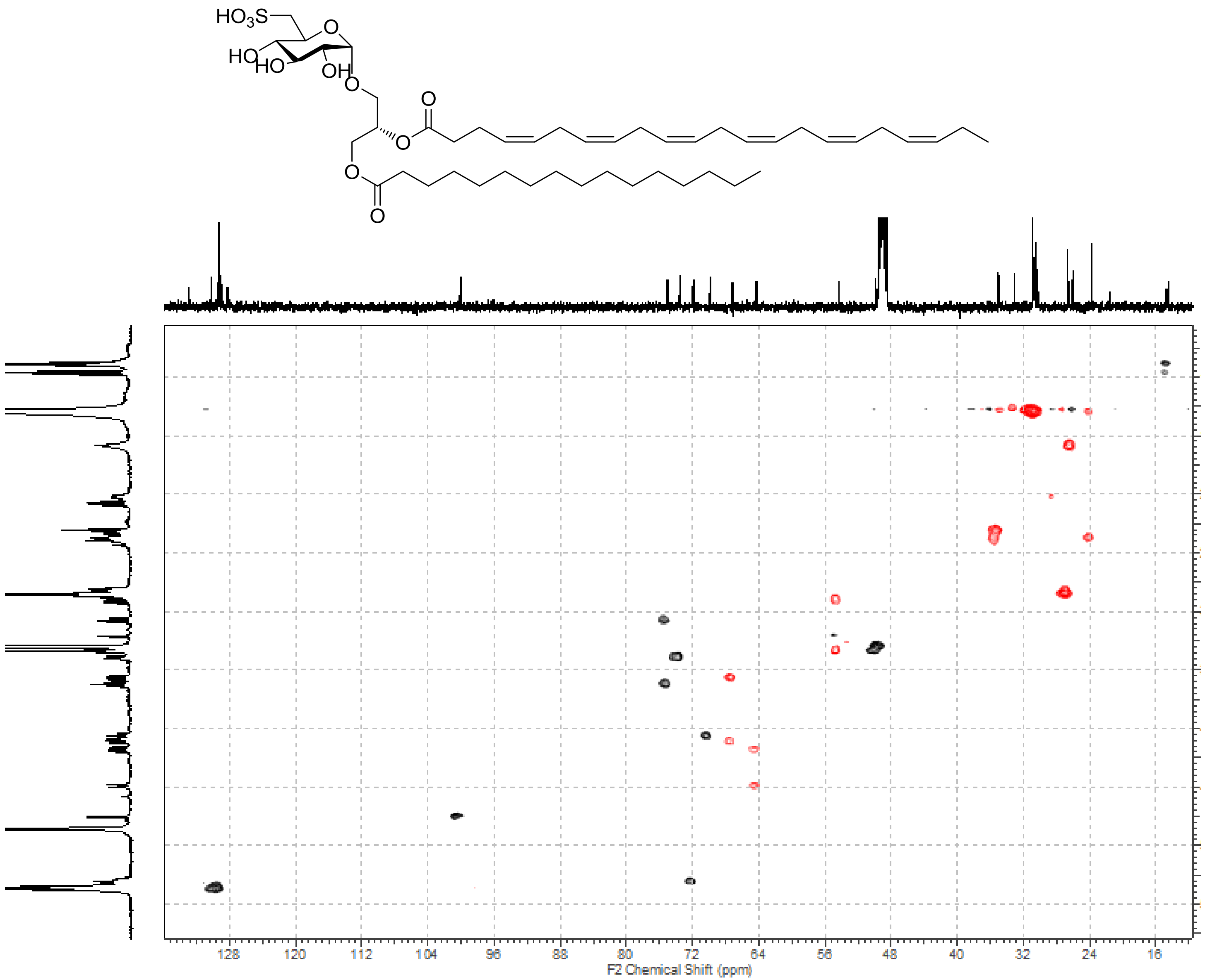

Figure S14. HSQC NMR spectrum of compound **2** at 500 MHz. (Black: CH, CH_3; Red: CH_2)

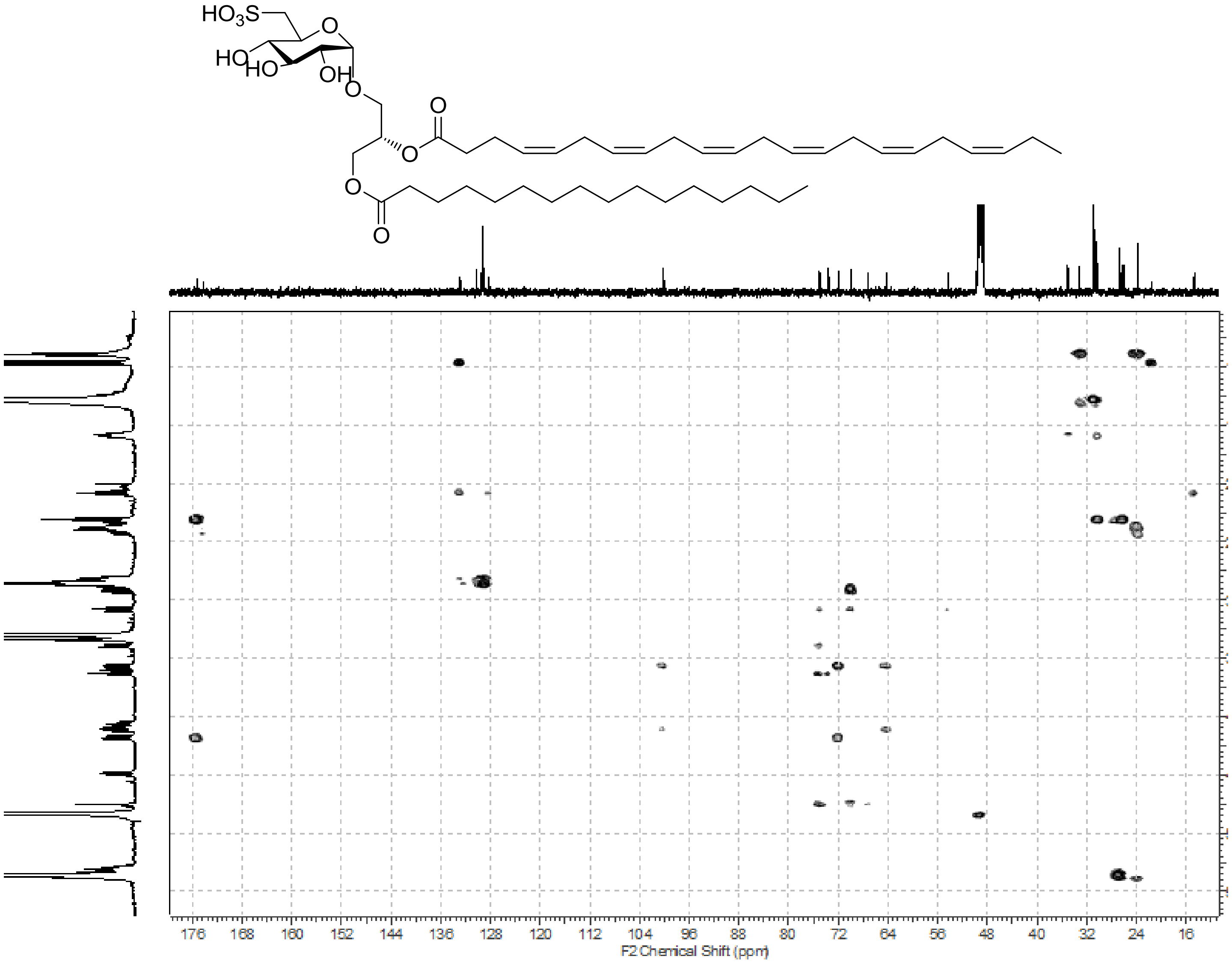

Figure S15. HMBC NMR spectrum of compound **2** at 500 MHz.

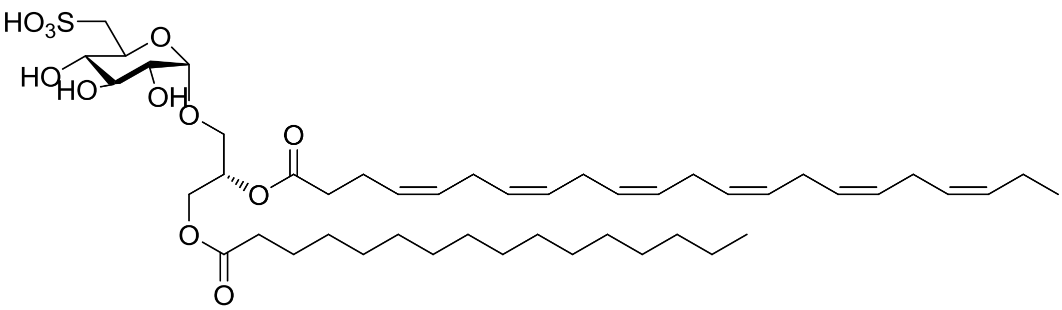

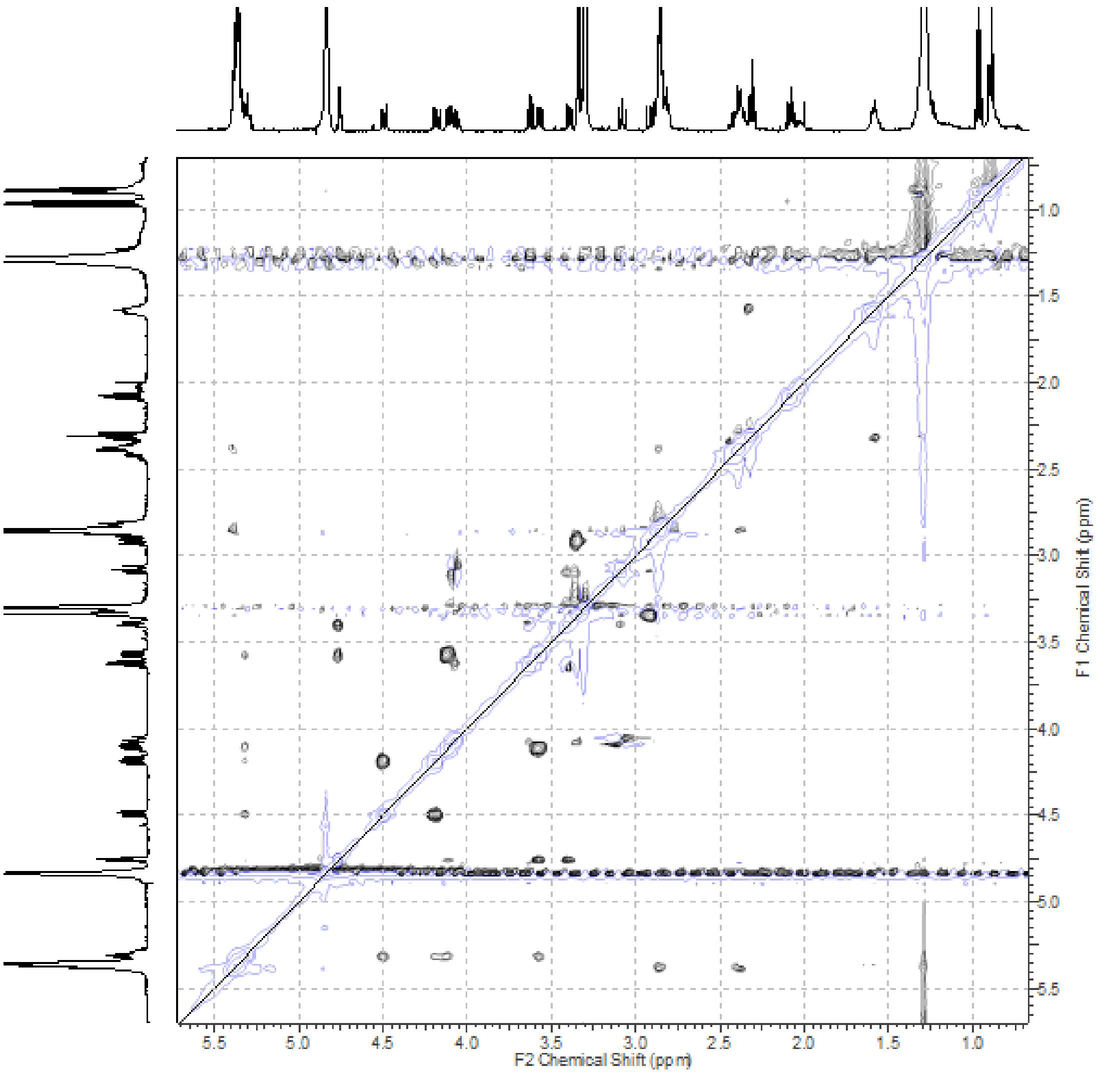

Figure S16. ROESY NMR spectrum of compound **2** at 500 MHz.
